# Assessment of the Nutritional Status, Diet and Intestinal Parasites in Hosted Saharawi Children

**DOI:** 10.3390/children7120264

**Published:** 2020-11-29

**Authors:** Mónica Gozalbo, Marisa Guillen, Silvia Taroncher-Ferrer, Susana Cifre, David Carmena, José M Soriano, María Trelis

**Affiliations:** 1 Area of Nutrition and Bromatology, Department of Preventive Medicine and Public Health, Food Sciences, Toxicology and Forensic Medicine, University of Valencia, 46100 Burjassot, Spain; 2 Area of Preventive Medicine and Public Health, Department of Preventive Medicine and Public Health, Food Sciences, Toxicology and Forensic Medicine, University of Valencia, 46010 Valencia, Spain; marisa.guillen@uv.es; 3 Clínica Universitària de Nutrició, Activitat física i Fisoteràpia (CUNAFF), Lluís Alcanyís Foundation-University of Valencia, 46010 Valencia, Spain; silvia.taroncher@fundacions.uv.es; 4 Parasites and Health Research Group, Department of Pharmacy and Pharmaceutical Technology and Parasitology, University of Valencia, 46100 Burjassot, Spain; sucimar@alumni.uv.es (S.C.); maria.trelis@uv.es (M.T.); 5 Parasitology Reference and Research Laboratory, National Centre for Microbiology, Carlos III Health Institute, 28222 Majadahonda, Madrid, Spain; dacarmena@isciii.es; 6 Observatory of Nutrition and Food Safety for Developing Countries, Food & Health Lab, Institute of Materials Science, University of Valencia, 46980 Paterna, Spain; jose.soriano@uv.es; 7 Joint Research Unit on Endocrinology, Nutrition and Clinical Dietetics, University of Valencia-Health Research Institute La Fe, 46026 Valencia, Spain

**Keywords:** Sahara, malnutrition, lactose malabsorption, celiac disease, intestinal parasites, hygiene, diet, health

## Abstract

Since the early 1990s, Spanish humanitarian associations have welcomed Saharawi children from the refugee camps in Tindouf (Argelia). These children are the most affected by the lack of food, water, hygienic measures and health care. The main objective of this study was to analyze the anthropometric, nutritional and parasitological data of 38 Saharawi boys and girls (from 10 to 13 years old) under a holiday host program in the city of Valencia. Our results confirm that malnutrition and multiparasitism are highly frequent, so it is understood that living conditions in refugee camps continue to be precarious with a lack of proper hygiene and nutrition. Furthermore, biochemical alterations, lactose malabsorption and the risk of celiac disease, also detected in our study as a secondary objective, will complicate nutritional management and restoration of health. For this reason, sustainable feeding alternatives and interventions from a hygienic and nutritional point of view are proposed, emphasizing in an improvement in the education of parents and children.

## 1. Introduction

Western Sahara, located in the African continent, is bordered in the north by Morocco, in the south by Mauritania, in the east by Algeria, and in the west by the Atlantic Ocean. Its population has suffered several episodes of military and political conflicts, which has led to refugee status. Refugee Saharawi families have been staying in the Tindouf (Algeria) camps for 43 years (since 1976), waiting for a political agreement allowing them to return to the Sahara. It was estimated that more than 40,000 Saharawis fled after conflict to Algerian camps (Laayoune, Auserd, Smara, Dajla, Rabunni and Bojador), and today around 180,000 Saharawis live there, according to the census of the United Nations High Commissioner for Refugees (UNHCR) and the government of the Saharawi Democratic Arab Republic (RASD). It should be noted that these camps distributed in the Algerian desert are located in one of the most inhospitable and arid areas of the world, with very harsh living conditions with a situation of total dependence on humanitarian aid. They lack vegetation and animals for their sustainability, as well as electrical installation and running water. Sirocco, violent and dry sandstorms, are characteristic of the place. The population lives in *jaimas* (canvas shops), in adobe houses and, in recent years, in cement constructions that have been built that include a latrine located outside the central unit. In the educational field, unprecedented success has been achieved moving from an illiteracy rate of 85% of the population to the total schooling of the children between 3 and 12 years with mandatory and free character [1]. With regard to health conditions in the camps, there are numerous factors with a direct negative impact on the health of the population: overcrowding, food and nutritional deficiencies, lack of water and in-unit medical care, and limited access to medicines. Food depends almost entirely on the help of international organizations that provide long-lasting staple foods, such as starch-rich foods and legumes, but few fresh vegetables and fruits, which is causing the double burden of malnutrition: overweight households versus those with malnutrition [2].

Regarding the general economic situation, it should be noted that it does not go beyond the stage of mere subsistence. As a people in exile, the population does not have the financial assistance of non-governmental institutions and humanitarian organizations, and its economic and productive activities focus on planting small orchards, raising some animals such as goats and workshops of textiles and traditional crafts. In the summer of 1979, thanks to the collaboration between the Front Polisario and the PCE (Communist Party of Spain), the first 100 Saharawi children arrived in Spain for a welcoming experience for Spanish families during the summer holidays. This program emerged so that Saharawi children could temporarily distance the very reality of refugee camps, the main objective being to provide good health care and treat diseases (both infectious and nutritional) during the months of stay. The experience was so beneficial that in the mid-1980s the “Holidays in Peace” program was launched, which consists of hosting a Saharawi child (7–13 years old) from the Saharawi Refugee Camp of Tindouf, during the months of July and August [3].

Several international projects have been carried out to collect anthropometric, nutritional and parasitological data of the Saharawi population, especially at-risk groups such as children and pregnant women. Most studies agree on the high prevalence of growth retardation and low weight [2,4,5,6,7]. Bilbao et al. [8] wrote a food guide called “*Food Jaima*” for host families in Spain about foods and the recommended daily amount, in order to improve their nutritional status. Other field implementation projects aimed to educate Saharawi mothers about dietary habits to make them protagonists of the improvements in the diet and nutrition of their sons and daughters [9].

On the other hand, data collection on intestinal parasitism in the Saharawi population, within the same host program, was carried out by Paricio-Talayero et al. [4], who observed that enteroparasites were present in 75% of the samples examined. Further studies were conducted over the years and confirmed that hygienic, personal and community measures remained insufficient for the control of parasitic infections and to prevent their long-term effects [4,5,6,10,11,12,13]. Medical examinations and antiparasitic campaigns were carried out in each of these studies through the administration of antiprotozoal and anthelmintic drugs during their stay in Spain. Most children had multiple infections, suggesting a general situation of lack of hygiene, food security and access to safe drinking water, in conjunction with children neglecting hygienic habits and the inability to generate an adequate immune response [12].

The aim of the present study is to verify if there have been changes with respect to previous studies on the parasitological, nutritional and health status of the hosted children in staying into the “Holidays in Peace” program, to design interventions that may improve their living conditions in the short and long term.

## 2. Materials and Methods

### 2.1. Design and Subjects of Study

This cross-sectional study was conducted with 38 Saharawi children: 16 boys and 22 girls (42.1% and 57.9%, respectively) with a mean age of 11.1 years (10–13 years), who were hosted in Valencia (Spain) during the summers of 2016 and 2017. The participants in this study were from the following Saharawi refugee camps: Smara (60.5%), Auserd (21.1%) and Laayoune (18.4%). For 60.5% of the participants, it was their first visit to Spain while the rest had already been to the country before. A percentage of 73.7% of the Saharawis interviewed had between 3 and 5 siblings and the place they occupied in their family was, in 55.3% of the cases, the first or the second, and in 31.6%, the third or the fourth. These were boys and girls who lived in generally large families, which, including parents, 58.4% of them had between 5 and 7 members. In 79.0% of the participants, they believed that their parents worked but did not know exactly what work they did.

The criteria for inclusion in the study were: to be a member of the host program and to obtain informed consent from Spanish families (as legal representatives) after being informed of the study and the data protection procedure. The intervention consisted of: (a) a physical and anthropometric examination; (b) a collection of personal data, sociodemographic variables (age, sex, camp of origin, number of siblings, professional occupation of parents), clinical and medical family history, frequency of consumption of food and hygienic habit as a risk factors for parasitic infection (hand washing, contact with animals, source of consumption water, etc.), all in the presence of a healthcare professional and with the help of a translator; (c) a coproparasitological analysis; (d) an analysis of lactose malabsorption using the breathed air test; € a determination of biochemical analytical variables related to nutritional status; and (f) a study of genetic markers associated with risk of celiac disease and primary lactose intolerance.

This study was approved by the Ethics Committee for Human Research of the University of Valencia (procedure number: HI48709978679, 2 March 2017) as part of the project “Promotion of hygienic-health habits for the prevention of intestinal parasites in the Saharawi population” ensuring that the fundamental principles established by the Helsinki Declaration and Spanish legislation in the field of biomedical research, data protection and bioethics are respected.

### 2.2. Anthropometric Assessment

The physical examination was performed by doctors from the Paediatrics Service of the University Hospital Clinic of Valencia (Spain) and was evaluated for the presence of lax skin, loss of muscle mass in limbs and buttocks, bloating and pubertal development. An Omrom^®^ electronic scale (accuracy, 100 g) and a Cescorf^®^ narrow and inextensible metal tape (accuracy, 1 mm) were used to obtain the weight and height. The height was determined, without shoes or socks, with a non-extensible tape measure, a blank sheet fixed on the wall and a carton; and body weight with participants dressed only in underwear. Height-for-Age and Body Mass Index-for-Age Z-scores (HAZ and BMIZ) were calculated using with WHO Anthro and AnthroPlus software (World Health Organization, 2009, Anthro for Personal Computers, Version 3.01: Software for Assessing Growth and Development of the World’s Children), using the WHO child growth standard 2005 version for children aged 0–5 years and the 2007 version for children and adolescents aged 5–19 years. These indicators classified children to varying degrees and types of malnutrition based on WHO reference data [14], which enable the analysis and interpretation of growth patterns of children of any population in the world.

### 2.3. Dietary Assessment

Data were collected on participants’ eating habits, such as the number of meals per day, schedules, food consumed and weekly frequency of consumption. For the assessment of the Saharawi child diet, the foods consumed were grouped together and the frequency of daily consumption of each was calculated. Dietary Diversity Score was determined using a modification of the food groups defined in FAO’s Minimum-Women’s Food Diversity (MDD-W) and the Food and Nutrition Technical Assistance Project [15]. The following food groups were considered: (1) Farinaceous (“All starchy foods”); (2) Legumes (“Beans and Peans”); (3) Dairy; (4) Protein foods (“Flesh foods”); (5) Vegetables and (6) Fruits (groups 5 and 6 corresponding to “Vitamin A-rich dark Green leafy vegetables,” “Other vitamin A-rich vegetables and fruits,” “Other vegetables” and “Other fruits”). It should be noted that this study did not take into account the consumption of vitamin A-rich vegetables, so this detail was obviated and only two groups were determined to collect the information these four points determined in FANTA [15]. With regard to the groups “Nuts and Seeds” and “Eggs,” they were not included because they were not consumed by the participating population. According to this indicator, consumption of at least 5 of the 10 established food groups indicates a higher likelihood of reaching micronutrient needs. In addition, the consumption frequencies obtained were compared to those recommended by Bilbao et al. [8] and Estruch et al. [16,17] and are able to assess their suitability.

### 2.4. Coproparasitological Assessment

For parasitological analysis, one sample of fresh stool per participant was taken. After the filtration and concentration of the samples, they were observed by optical microscopy for the identification of forms of resistance of intestinal parasites in general (cysts or eggs); molecular diagnostic techniques (conventional and real-time polymerase chain reaction or qPCR) were also applied to some specific parasites (*Giardia intestinalis* and *Blastocystis* sp.).

Starting from 3 g of fecal sample, they were filtered and concentrated by centrifugation (2500 rpm, 5 min) in Midi Parasep tubes^®^ (Apacor Ltd., Wokingham, UK). The sediment obtained was divided into two microtubes, one for extraction of total stool DNA for qPCR, and the other with 10% formalin for microscopic observation. The QIAmp DNA Stool Mini Kit (QIAGEN^®^, Hilden, Germany) was used for DNA extraction according to the manufacturer’s instructions.

For the diagnosis of *G. intestinalis* DNA, a qPCR was performed. This protocol specifically amplifies a fragment of the gene that encodes the parasite’s small ribosomal subunit RNA (*SSU* rRNA) showing high sensitivity [18]. A commercial assay with the specific primers and probe (LightMix Modular Assays Giardia, Roche^®^, Basel, Switzerland) was used, together with the mastermix (dNTPS, thermostable Taq polymerase and buffer) (PerfeCTa qPCR ToughMix, Quanta Biosciences, Gaithersburg, MD, USA) for a mix reaction final volume of 15 μL. To each well, 5 μL of sample and positive control DNA was added, and instead water for negative control. The analysis was performed with the StepOne Plus real-time PCR thermocycler^®^ (Applied Biosystems^®^, Foster City, CA, USA). Any sample that manages to amplify before 43 cycles was considered positive.

Positive samples for *G. intestinalis* by qPCR were subsequently analyzed by multilocus genotyping based on the sequences of genes encoding the parasite’s glutamate dehydrogenase (*gdh*) and β-giardine (*bg*) proteins [19]. A semi-nested PCR protocol proposed by Read et al. [20] with minor modifications was used to amplify a fragment of 432 bp of the *gdh* gene with the GDHeF/GDHiR primer pair in the primary reaction and GDHiF/GDHiR in secondary. In parallel, a fragment of 511 bp of the *bg* gene of *G. intestinalis* was amplified using the nested PCR protocol described by Lalle et al. [21] with G7F/G759R primers in the primary reaction and G99F/G609R in secondary [21]. In the case of *Blastocystis* sp., a direct PCR assay was used, targeting the *SSU* rRNA gene using the *Blastocystis* barcode primer pair RD5/BhRDr to amplify a product of 600 bp as previously described [19,22].

All the direct, semi-nested and nested PCR protocols described above were conducted on a 2720 thermal cycler (Applied Biosystems^®^). Reaction mixes always included 2.5 units of MyTAQ—DNA polymerase (Bioline^®^, London, UK), and 5 MyTAQ-Reaction Buffer containing 5 mM dNTPs and 15 mM MgCl_2_. Laboratory-confirmed positive and negative DNA isolates for each parasitic species investigated were routinely used as controls and included in each round of PCR. PCR amplicons were visualized on 2% agarose gels stained with SafeView Nucleic Acid Stain (NBS Biologicals^®^, Huntingdon, UK).

Positive-PCR products were directly sequenced in both directions using the internal primer set described above. DNA sequencing was conducted by capillary electrophoresis using the BigDye^®^ Terminator chemistry (Applied Biosystems^®^, USA) on an ABI PRISM 3130 automated DNA sequencer. Raw sequencing data in both directions, back and forth, was determined using the Chromas Lite version 2.1 sequence analysis program (https://technelysium.com.au/wp/chromas/). The BLAST tool (http://blast.ncbi.nlm.nih.Gov/Blast.cgi) was used to compare nucleotide sequences with sequences retrieved from the NCBI GenBank database. Finally, generated sequences samples were aligned with appropriate reference sequences using MEGA6: Molecular Evolutionary Genetics Analysis Version 6.0 [23] software to identify *G. intestinalis* assemblages and sub-assemblages. Generated *Blastocystis* sp. sequences were submitted to the publicly available database *Blastocystis* 18S (http://pubmlst.org/blastocystis/) for subtype confirmation and allele calling [23]. The sequences generated in this study have been deposited in GenBank under accession numbers MW265975-MW265987 (*G. intestinalis*) and MW265003-MW265005 (*Blastocystis* sp.).

### 2.5. Laboratory Evaluations

Fasting venous blood samples were obtained from each of the participants for the determination of blood cells, leukocytes, leukocyte formula and platelets. Glucose, creatinine, urea, transaminase (GOT and GPT), alkaline phosphatase, LDH, Immunoglobulins (A, G and E), anti-transglutaminase and anti-endomysium antibodies were also analyzed [24].

The method used for the diagnosis of lactose malabsorption is called “Expired air test” or “Breathtracker gas chromatograph” (QuinTron Instrument Company Inc., Milwaukee, WI, USA), which allows the measurement of hydrogen (H_2_) and methane (CH_4_) levels contained in the exhaled air after the intake of a sugary substrate. These gases are volatile compounds formed from the bacterial colonic fermentation of sugars that remain in the gut due to their incomplete digestion. To prepare the test, food should be taken in normal quantity the day before, but by restricting the intake of carbohydrates and sugars, the patient should also be fasting for at least the previous 8 h (ideally 12 h) and can only drink water until 4 h before performing it. At the beginning of the test, the patient must breathe into the apparatus to obtain the “basal” gases (ppm) data. The patient then takes an aqueous solution of lactose (25 g of lactose dissolved in 250 mL of water). After that, samples of breathed air are taken every half hour (30, 60, 90, 120, 150, 180, 210 and 240 min) to measure the evolution of the gas content. The “basal” level of H_2_ must be less than 10 ppm, and less than 6–8 ppm for CH_4_, for the test to be valid and consider its production to be related to the administered substrate. The test is positive when, in any of the measurement points before minute 90, the 20 ppm in hydrogen is exceeded with respect to the “basal” value or the lower of the previous ones, and between 10–15 ppm in the case of methane [25,26].

For the genetic analysis of risk of celiac disease and primary lactose intolerance, were carried out in a small representation of the studied population, oral mucosa samples were obtained using a sterile swab and analyzed in Overgenes S.L. laboratory (Valencia, Spain) for analysis. The presence of celiac disease risk markers (HLA-DQA1 and HLA-DQB1 alleles) were determined. Regarding primary lactose intolerance, five genetic single nucleotide polymorphisms (SNPs) associated with the persistent lactase phenotype were analyzed, two of them more frequent in Caucasian populations and the other three in populations of African origin, all of them present in the sequence of the MCM6 gene, known to regulate lactase expression. The risk genotypes for the 5 SNPs of the MCM6 gene associated with primary lactose intolerance analyzed were: C/T_13910, G/A_22018, G/C_14010, T/G_13915 o G/C_13915 and C/G_13907, which determine the persistent lactose status [7,24,27,28].

### 2.6. Statistical Analysis

Descriptive statistics were calculated including measures of central tendency (mean and median), measures of dispersion (standard deviation, range and coefficient of variation) and measures of shape (asymmetry and pointing) for quantitative variables, as well as the absolute and relative frequencies for the qualitative variables. Association analyses were performed stratifying by sex and age to observe possible heterogeneity in the results according to these factors. Due to the sample size, non-parametric tests were used (Fisher’s exact test and Mann-Whitney U test). Any p value less than 0.05 was considered as statistically significant. All the variables were analyzed using SPSS software (Statistical Package for Social Sciences for Windows, version 26.0, SPSS Inc., Chicago, IL, USA).

## 3. Results

### 3.1. Anthropometric Assessment

Table 1 shows HAZ and BMIZ values from Saharawi children. In comparison with WHO standards [14,29,30,31], HAZ data reflected moderate and severe chronic malnutrition (or stunting) in 10.5% and 5.3%, respectively, of the studied children, and 28.9% of them had increased risk of child stunting. In fact, high presence of chronic malnutrition (with impaired height and even lower cognitive development) could be explained by the lack of an adequate, balanced and sufficient diet for prolonged periods, as well as recurrent infections that hinder progression towards normal growth values. The data were analyzed stratifying by sex, but no statistically significant differences (*p* > 0.05) were found for any of the variables.

**Figure 1:**
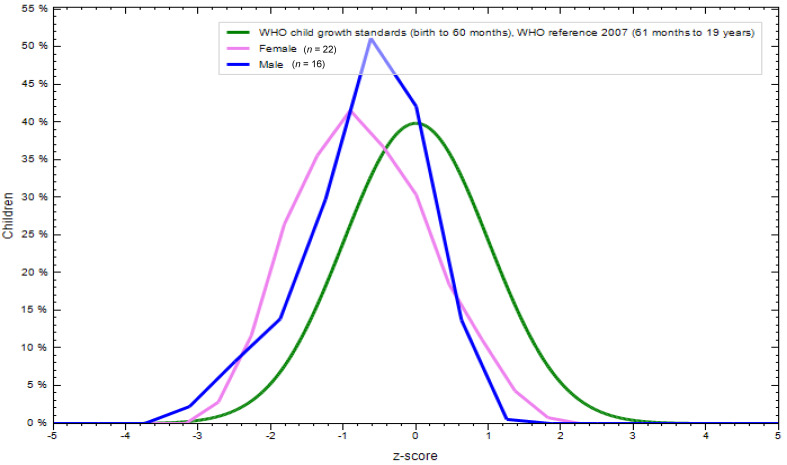
Body Mass Index-for-Age Z-scores (BMIZ) distribution of the Saharawi population (blue: male children; purple: female children; green: World Health Organization (WHO) reference standard).

For BMIZ, the characteristics of malnutrition are characterized by insufficient intake combined with nutrient malabsorption by infectious factors or recurrent diseases. The prevalence of mild and moderate malnutrition as defined was observed in 31.6% and 5.3%, respectively. No statistically significant differences were found when the distribution of the BMIZ by sex was analyzed. However, if obtained results were stratified by age, it was observed that the group with the highest risk of mild malnutrition was that of 10 years of age (4 girls and 3 boys) (70% vs. 6.7% in 11 years, 27.3% in 12 years and 50% in 13 years; chi^2^ = 22.296; *p* = 0.001). The joint assessment of the BMIZ of all Saharawi boys and girls is represented in Figure 1 as a Gaussian curve with a standard deviation of 0.75 and with an average of −0.6. This curve is displaced to the left (noir-colored line) compared to what would be a normal distribution (gray-colored line). The conclusion that is drawn is that a significant percentage of children are in negative values, with a risk of emaciation, presenting an impairment of growth in both weight and height with respect to the reference values. Therefore, actions should be aimed at reducing the average as close to zero as possible.

### 3.2. Dietary Assessment

The analysis of the information collected in the surveys showed that all the participants ate between 3 and 4 meals per day. Regarding the times of these meals, 84.6% had breakfast between 7:00 and 8:00 a.m., 76.9% claimed to eat between 1:00 p.m. and 1:30 p.m. For snacks, 75.1% had it between 5:00 p.m. and 6:00 p.m.; 46.2% of the participants commented on having dinner between 9:30 p.m. and 10 p.m.; and 38.5% declared to carry out late night snack. The main foods consumed by the Saharawi child population were those known as basic foods, such as rice (97.4%), couscous (86.8%), milk (81.6%) or lentils (86.8%), but other foods, with low nutritional value, were occasionally consumed including biscuits, chocolate, Coke beverage and candy (Table 2).

According to the classification of food group from FAO FANTA project [14], an average daily consumption frequency of 2.78 ± 1.82, 0.96 ± 0.87, 2.38 ± 1.96, 0.80 ± 0.70 and 1.02 ± 0.67 servings of farinaceous, legumes, dairy products, fruits and protein per day, respectively, were observed in our study. Surprisingly, vegetables were not eaten by any children. Number of consumed food groups is shown in Table 3.

A percentage of 7.9% consumed foods from a single food group, 13.2% consumed from 3 and 5 food groups, 18.4% from 2 food groups, and practically half of the children (47.4%) consumed foods from 4 food groups. No participant consumed at least 5 food groups to ensure correct micronutrient needs. No significant differences were obtained in the consumption of the different food groups between sexes. Furthermore, our group asked about the possibility that some foods caused them gastrointestinal problems (vomiting and/or diarrhea) and found that 47.1% experienced such, with milk (37.5%) and fruit (12.5%) as the causative food.

On the other hand, all the children participating in the study declared to always wash their hands before going to eat and after going to the bathroom. Furthermore, 87.5% of the children declare that they brush their teeth and, when asked about the frequency of brushing, 37.5% do it once a day, 12.5% twice a day and 37.5% three times a day or more. When the frequency of toothbrushing stratified by sex was analyzed, no statistically significant differences were observed between boys and girls in tooth brushing. A percentage of 56.3% of the children declared that they had animals at home, of which 55.5% are cats, 33.3% are goats and 11.1% claimed to have contact with dogs.

### 3.3. Coproparasitological Evaluation

Results of the parasitological analysis are shown in Table 4. Overall, 97.4% of the participants presented intestinal parasites. Of them, 97.4% of parasitization was by protists and 26.3% by helminths (the prevalence of combined protist-helminth infection was also 26.3%). Multiparasitism was the most frequent, highlighting 5.3% of cases harboring seven enteroparasitic species. The most prevalent intestinal protist parasites were, from highest to lowest: *G. intestinalis* (92.1%), *Blastocystis* sp. (86.8%), *Endolimax nana* (52.6%), *Entamoeba coli* (47.4%), *Entamoeba hartmanni* (36.8%), *E. histolytica/E. dispar*/*E. moshkovskii*/*E. bangladeshi* (13.2%), *Iodamoeba butschlii* (13.2%) and *Dientamoeba fragilis* (5.3%). Regarding helminths, only two species were detected, *Hymenolepis nana* (18.4%) and *Enterobius vermicularis* (10.5%). Most of these parasitic species are ingested as cysts or eggs (infectious forms) present in food or water by fecal contamination. No statistically significant differences were obtained when stratifying by sex.

The overall prevalence of *G. intestinalis* and *Blastocystis* sp. varied according to the method employed. By direct diagnosis (light microscopy), the occurrence of both species was estimated at 52.6% and 71.1%, respectively. In addition, a prevalence of 60.5% for *G. intestinalis* was obtained by indirect ELISA using anti-*Giardia* (IgA) antibodies, and 81.6% by qPCR. The latter prevalence was also obtained for *Blastocystis* sp. by conventional PCR. Mixed infections involving these two parasitic species were common (81.6%) in the studied Saharawi population. No association analysis could be performed between nutritional status and the presence of intestinal parasites since the entire sample was infected by one or more parasite species.

Furthermore, a total of 31 DNA isolates were positive for *G. intestinalis* by qPCR, providing Ct values ranging from 19.0 to 37.0 (median: 26.3). Of them, 25.8% (8/31) and 16.1% (5/31) were successfully amplified at the *gdh* and *bg* loci, respectively. A total of eight isolates were genotyped and/or sub-genotyped for either of the two markers. Multilocus genotyping data were available for 25.8% (8/31) of the characterized isolates. Only seven *gdh* and six *bg* PCR amplicons were obtained from *G. intestinalis* isolates, highlighting that the low amplification rates obtained for both markers were highly dependent on the Ct qPCR values. Table 5 shows the diversity, frequency and main characteristics of the *G. intestinalis* sequences generated at the *gdh* and *bg* loci.

Sequence analysis revealed the presence of assemblages A (25.0%; 2/8) and B (75.0%; 6/8). Canine (C, D), feline (F) or ruminant (E) assemblages were not detected. Of the two assemblage A sequences, one was assigned to the sub-assemblage AII at the gdh locus and the remaining showed an ambiguous AI/AII result when the two *gdh* and *bg* loci were considered. Similarly, the sequence analysis of the six isolates assigned to assemblage B allowed the identification of sub-assemblage BIII (37.5%; 3/8) and BIV (12.8%; 1/8). Ambiguous BIII/BIV results were determined in 25.0% (2/8) of the isolates at the *gdh* locus. The sequences of the genotyped parasites have been also deposited in GenBank for subsequent molecular epidemiological studies in the future (Table 5).

Regarding the molecular characterization of *Blastocystis* sp., from the 31 positive isolates by PCR, 41.9% were successfully subtyped by sequence analysis in the *SSU* rDNA gene. BLAST searches allowed the identification of three subtypes, including ST1 (46.2%; 6/13), ST2 (7.7%; 1/13), and ST3 (42.6%; 6/13). Neither mixed infections involving different STs of the parasite nor infections caused by animal-specific ST10-ST17, ST21 or ST23-26 were recorded.

### 3.4. Analytical Test Results

The most frequent analytical alterations occurred at the enzymological level in transaminases (ALAT and ASAT), alkaline phosphatase (ALP) and lactate dehydrogenase (LDH). All those that showed high values of any of them (5.3%, 10.5% and 2.6%, respectively) were parasitized by *G. intestinalis*. Although no association of any of them with parasitosis has been reported, there is an association of increased transaminases with cases of untreated celiac disease.

For determinations of total antibodies, immunoglobulins IgG and IgE were elevated in 5.3% and 2.6% of the studied population, respectively. High values of IgG could be a consequence of natural immunization state or vaccination, while elevated IgE is used to be related to infections with intestinal parasites and allergic reactions.

Regarding hematic alterations, for mononuclear leukocytes, it showed high values in terms of monocytosis (7.9% of cases) and lymphocytosis (15.8%). As for polynuclear leukocytes, eosinophilia was detected in 15.8% and neutropenia in 13%. In relation to the red series, an erythrocytic count decreased by 21.1%, with low plasma concentrations of ferritin in 5.3%, of haemoglobin in 21.1% and serum iron in 5.3% of the participants, and, finally, highlighted an increased platelet count in 10.5% of the cases. When the analysis of the association between haematological alterations and nutritional status (HAZ and BMIZ) was carried out, no statistically significant results were obtained for any of the variables.

Lactose malabsorption test was positive in 26.3% of the studied children, 12.5% and 36.4% for male and female, respectively, but without significant differences between sexes. The association between lactose malabsorption and nutritional status assessed by the BMIZ was analyzed, and no statistically significant result was found. However, it was observed in malabsorbers a BMIZ (15.9 ± 1.5 kg/m^2^) lower than in absorbers (16.1 ± 1.5 kg/m^2^) without significant differences. On the other hand, there was no association between lactose malabsorption state and eating habits, especially with the group of dairy products and derivatives, and no reduction in intake was observed in malabsorbers. Of the participants, 97.4% regularly ingested dairy products and derivatives, although 18.4% reported digestive discomfort after consuming them. Malabsorption could be a cause of intolerance and rejection of these foods. Furthermore, two of them (5.2%), coinciding with the ones with digestive disorders, shared lactose malabsorption and genetic risk of celiac disease.

According to the results obtained about lactase activity in the Saharawi population, by the expired air test measuring the intestinal capacity of absorption of the sugar, the prevalence of children with malabsorption was 26.3%. Meanwhile, by determination of genetic predisposition to develop a deficiency of the enzyme, 50% of the group of children analyzed showed an absence of the protective five polymorphisms of the lactase gene (MCM6).

For determination of the genetic risk of celiac disease, genes HLA-DQ8B1 and HLA-DQA1 were analyzed. A percentage of 62.5% of the genetically studied Saharawi population presented genetic (haplotype) combinations associated with the risk of developing celiac disease, with no significant differences between sexes. The predominant phenotypes detected were: DQ2.2/DQ8 (high risk) (25%), DQ2.2/DQ-(moderate high risk) (12.5%) and DQ8/DQ-(moderate risk) (25%). The association between the risk of developing celiac disease and the nutritional status assessed through the BMIZ was analyzed and no statistically significant results were found. However, it was observed that those at risk had a BMIZ of 15.5 ± 0.7 kg/m^2^, lower than that of children who were not at risk, whose BMIZ was 16.1 ± 1.6 kg/m^2^ (*p* > 0.05). According to eating habits, those with a predisposition to celiac disease reported consuming less than 1 time/day of food from the farinaceous group, specifically bread or pasta, although without statistical significance.

## 4. Discussion

Saharawi children are a population that, due to a series of cultural, social and political characteristics, have a high probability of suffering parasitic diseases. These parasites are a problem in developing countries, since they are generally underestimated for being asymptomatic, but they represent an important morbidity factor when associated with malnutrition [32]. Furthermore, it is interesting to reflect that our study has a series of limitations that occurred during data collection from the participating children. The collection of the anamnesis was very difficult not only because of the ignorance of their previous pathologies, but also because of the difference in language and the young age of the girls and boys, and even the difference in customs made during data collection, but the use of interpreter was very helpful. For the physical examination, the children were extremely modest since the factor of age is added to the mistrust generated by another cultural environment, making it convenient that they be assessed by a pediatrician of the same sex. The collection of feces for parasite testing was particularly problematic for host families due to the frequent refusal of children.

For the physical examination and the collected anthropometric data, our study demonstrated that the children, being in the growth and development stage, were, in general, included in Z-scores ≥−2 SD and <+1 SD (between normal weight and risk of chronic malnutrition) according to the growth indicators of BMIZ. The WHO Growth Pattern confirmed that all children born anywhere in the world who receive optimal care from the beginning of their lives have the potential to develop in the same range of height and weights. Of course, there are individual differences between boys and girls, but regionally and globally, the average population growth is remarkably similar. The pattern shows that differences in child growth depend more on nutrition, feeding practices, environment and health care than on generic or ethnic factors. The WHO Child Growth Standards have worldwide validity. Its purpose is to monitor the growth of all children throughout the world regardless of their ethnicity, socioeconomic status and type of diet [14]. Thus, of the study participants, only two children (a boy and a girl) exceeded this limit due to a weight deficit. According to the HAZ, a high presence of low stature was determined (six children in total), a fact that could be explained considering that the sample studied, whose mean age was 11.1 years, had been lacking adequate nutrition for many years, thus presenting a chronic malnutrition that at the same time increases the probabilities of infections by parasites, in particular, which hinders the progression towards normal growth values. It should be noted that the results of this study are similar to those observed in previous studies of this population that showed that chronic malnutrition is frequent (percentages between 20% and 33%) [2,4,5,6].

According to eating habits, it is worth highlighting the data obtained in the study on the diet of Saharawi boys and girls with respect to the high consumption of sugary drinks, specifically Coke beverage, which 44.7% claim to consume daily, and of candy, which in this case, the percentage rises to 55.2%. This is in contrast with the Aladino study where 12.1% of Spanish children indicate consuming them between 1 and 3 days/week [33]. Leone et al. [34] observed in a Saharawi adult female population that the consumption of sugary beverages was by 62.5% of those surveyed, so the adult population is not a good reference of children towards this beverage consumption. On the other hand, observing the low contribution of fruits, it is common to think about possible deficiencies of vitamins and micronutrients, so increasing the consumption of these foods is important to reverse the high rates of anemia observed in this population [4,5,6,12,35]. Surprisingly, the consumption of vegetables is null, suggesting that they may not have any type of crop due to the aridity of the land or even a limited supply. It can also be observed that the consumption of oil (olive, sunflower, etc.) and nuts is infrequent, which can be considered as a deficiency of monounsaturated and polyunsaturated fatty acids. Furthermore, if energy inputs and fiber are reduced, it is reflected in the anthropometric assessment. Leone et al. [34] determined that the consumption of vegetables is frequent, although the variety of vegetables consumed is low (tomatoes, onions and carrots represent 90%). Further, sunflower oil is the most consumed, but it is noteworthy that only peanuts, from the nut groups, are part of their diet, and cereals and derivatives are consumed by 50% of those surveyed. If our data are compared with the food Jaima [8], it can be seen that it is an inverted style. Foods that should be at the base of the Jaima are at the peak, with very low or almost zero consumption, as is the case of fruits and vegetables, while sugary foods or drinks, which should be consumed occasionally, occupy the base of the pyramid with a very high consumption. It is highlighted that the number of food groups consumed by the participating children, according to the FANTA [15] distribution, is similar to the study carried out by Morseth et al. [36], in which aspects related to the dietary diversity of Saharawi refugees were evaluated, although the study comprised adults aged between 18 and 82 years. Therefore, it is necessary to provide tools that allow the development of a population (both children and adults), promote personal and work growth, facilitate access to education and better living conditions, as well as increase the availability and variety of food, facilitate food education and minimize nutritional deficiencies.

For hygienic-sanitary habits, it should be mentioned that children know very well what the appropriate response is, although the reality is different, so it can be observed that 100% of children say they wash their hands before eating and after going to the bathroom, when they do not even have enough running water to carry out these tasks on a regular basis. Hygienic measures are considered a key preventive measure against oral-fecal infectious diseases. Washing your hands after using the bathroom and before eating or preparing a meal can reduce childhood diarrhea. A report published by UNICEF [37] revealed that global rates of hand washing with soap and water before and after using the bathroom or handling food vary between 0% and 24%, which concluded that a significant part of the world’s population does not comply with basic hygiene measures, with children being the most vulnerable group.

Parasitic intestinal infections mainly affect the child population, which is the most susceptible to the development of acute symptoms, especially when the infectious form penetrates orally. Even at these ages, reinfections are usually more frequent than in adulthood, where parasitic infections are usually chronic [32,38]. The results of the coproparasitological analysis stand out from those obtained in other studies carried out in the same area or with children sheltered in different places in Spain [4,5,6,7,10,11,12,13]. In this study, higher prevalence of parasites is observed in general, although it should be noted that the most prevalent parasitic species are the same in practically all studies. According to Paricio-Talayero et al. [4] and Martínez and Pérez [11], *G. intestinalis* in 17.6% and 13.5%, *Blastocystis* sp. in 21.8% and 24%, and *E. nana* in 17.1% and 2.7%, respectively, were determined in host children in Spain, while among helminths: *H. nana* in 10.6% and 24%, and *E. vermicularis* in 4.7% and 35%, respectively, were determined in the same mentioned studies. In the two studies carried out in the field (Tindouf) [5,6], prevalence of parasites by protists of 60% and 45% were obtained, respectively, while that in the studies of Sarquella et al. [10] and Seseña del Olmo et al. [13] were determined as a total 19.6% and 37.3% parasitization, respectively. It is worth mentioning the fact that there is a high degree of intestinal parasitization by *G. intestinalis* and *Blastocystis* sp., maybe due to the use of different coprodiagnosis techniques in this study that had complemented the information. Since the emission of cysts along with the feces seems to be intermittent, microscopic diagnosis as a gold standard technique is not effective; therefore, the combination with molecular methods (such as PCR) that evaluate the intestinal presence of a certain parasite improve the diagnostic sensitivity [38]. Furthermore, the origin of the high prevalence of both protists can be due to poor health of drinking water [39] and the hygienic-sanitary habits (it is usually eaten from the same container with the hands), which facilitates oral-fecal transmission [40]. In addition, these parasitic infections, which are often asymptomatic [32], may be a trigger for secondary food malabsorption/intolerance (in particular, to carbohydrates), dyspepsia or irritable bowel syndrome [38,41,42,43,44]. Moreover, the alterations that they cause are related to cases of anemia, acute and chronic diarrhea and malabsorption syndrome can be explained by the presence of *G. intestinalis*. This parasitic protozoan is known to cause the activation of CD8 lymphocytes in the intestinal villi in the absorbent mucosa, which affects the growth and cognitive development of the child affected population [43,45]. Recent studies have associated this protozoan with alterations in iron absorption, decreased serum iron, low levels of Hb in the blood and, in general, with iron deficiency anemia [33,45,46,47,48,49]. Iron is absorbed in the duodenum, specifically in the enterocytes, and the mechanism of action of *G. intestinalis* is to line the intestinal mucosa and prevent the absorption of this mineral. Malnutrition, due to a diet that is not balanced or varied, is the result of the living conditions to which they are subjected. Therefore, this is the cause that affects the Saharawi, iron deficiency due to a diet deficient in this micronutrient. Host parents are advised to intensify their intake of iron-rich foods as well as oral iron supplementation if necessary. Serum iron, transferrin saturation index (TSI) and decreased ferritin appear in these children.

Human giardiasis is considered a zoonotic infection. The molecular characterization of *G. intestinalis* in the recent years has revealed significant genetic diversity. Assemblages from A to H have been identified, with assemblages A and B being the most diverse with at least four types of sub-assemblages (AI-AIII and BIII-BIV). Assemblages A and B are the most common in humans and have also been documented in wildlife and domestic animals. Assemblages C and D are found in dogs, and assemblages E, F and G in domestic ruminants, cats and rodents respectively, while assembly H has been described in marine pinnipeds [50]. *Blastocystis* sp. can remain in the body without causing symptoms but secretes proteases that are the cause of abdominal spasms, vomiting and diarrhea, with the consequent malabsorption of nutrients and the rejection of certain types of food [42]. There are hypotheses that support that this variation in host symptoms is related to the *Blastocystis* subtype [44,51,52]. Presently, 10 subtypes (ST1-ST9 and ST12) have been found in humans, 4 of which are the most frequently found: ST1, ST2, ST3 and ST4, coinciding with those determined in the participating Saharawi population. In addition, various studies attribute *Blastocystis* sp. controversial pathogenicity, even suggesting that the native microbiota or the immune status of the host may determine the pathogenicity and virulence of the protist [44,50,51].

On the other hand, *E. histolytica* destroys tissues through adherence to cells, leading to amoebic colitis and acute diarrhea, anorexia and low weight. *Entamoeba hartmanni, E. nana* or *E. coli*, although they have less relevance from the clinical point of view, may be useful indicators of poor personal and community hygienic-sanitary conditions in the population studied (especially in children) and its environment, as they are also transmitted through the fecal-oral route [53]. For helminth infections, they also produce nutrient malabsorption, and the relationship between anemia and the presence of helminths is frequently observed. Therefore, it is associated with an altered nutritional and cognitive status [54,55]. Finally, multiple infections or multiparasitism may be due to previous malnutrition, differences in children’s hygienic-sanitary behavior, the irregular distribution of infective stages in the environment, differences in the ability to generate an adequate immune response to basic differences between parasites and, also, genetic differences between hosts [12].

The participants underwent a blood test that estimated low levels of some indicators of anemia such as haemoglobin, haematocrit and red blood cell count, and also highlighted the presence of other values such as high levels of eosinophils and monocytes, indicators of parasitic infections and low levels of IgA, which favors intestinal colonization. These indicators were altered in previous studies in the refugee population and are related to signs of malnutrition in those parasitized, in particular, with *G. intestinalis* [5,35,47,56].

The breath test, despite being a non-invasive test and considered the reference method for the diagnosis of carbohydrate malabsorption, has its limitations [25,57]. In our study, more than a quarter of the host children were positive to the secondary lactose malabsorption/intolerance, and half of the children analyzed had primary lactose intolerance. Naturally after weaning, and depending on race, ethnicity and genetics, lactase activity persists or does not for a longer or shorter period of time, also depending on the lactose that is ingested. In cases of absent or reduced synthesis of lactase, this substrate reaches the colon where the bacteria metabolize it, generating abdominal pain or diarrhea. The relationship between symptoms and diet in patients with functional digestive disorders may lead to a suppression of dairy consumption, with the risk of a calcium and vitamin D deficiency in children [28]. Some of the Saharawi children studied reported abdominal pain after ingestion of milk. However, a history of post-ingestion symptoms is of little use in determining that a patient has lactase deficiency or lactase malabsorption [58]. Chaud et al. [59] obtained 90.8% of lactose malabsorption of the parasitized by *G. intestinalis*, compared to 7.5% in the control group. More studies are needed to link carbohydrate malabsorption in the general population, and in childhood and adolescence, in particular, with intestinal parasitization, which is not well documented. Another study in African and Finnish children suggested that decreased enzyme activity may be earlier in African children [60]. The study carried out by Rollán et al. [28] determined a high frequency of lactase deficiency associated with the C/T_13910 polymorphism of the MCM6 gene, as well as a high diagnostic yield of the genetic test, comparable or superior to the expiratory test, as observed in the data obtained in the Saharawi child population.

To detect celiac disease, two types of tests are performed to diagnose it: serological tests and genetic tests. Serological samples are focused for high levels of certain antibodies in the blood that reveal an immune reaction triggered after the consumption of foods containing gluten, although it should be noted that a negative result does not totally rule out the existence of celiac disease. On exposure to gluten, plasma cells produce IgA class anti-human tissue transglutaminase antibodies (IgA-TG2) and deaminated peptides (IgA-DGP70 and IgA-DGP71) [24,61]. In our analyzed blood samples, some of the participants had IgA-TG2, although the interpretation of the results of the genetic predictive test for celiac disease did not reveal the probability of suffering from the disease, nor were they statistically significant to establish any relationship. Regarding genetic tests, the presence of certain HLA-DQ heterodimers and their association were detected, which indicated a risk of suffering from the disease in 62.5% of the analyzed participants, and that they can make them more susceptible to intestinal parasites due to the damage caused to the intestinal mucosa. Previous studies suggest that gluten sensitivity is one of the common disorders in North Africa and the Eastern Mediterranean, determining a high frequency of celiac disease in the Saharawi population, and its consequent delay in physical and mental growth [10,62,63,64,65,66].

## 5. Conclusions

Low height was in approximately one-fifth of the children in foster care, and the prevalence of parasites was almost 100%, with multiparasitic infections being the most frequent. It should be noted that intestinal parasitosis is a contributing factor to malnutrition and iron deficiency; therefore, it is common to find problems such as anemia, celiac disease and growth retardation among the child population. The diet of the Saharawi children in the camp is deficient in terms of quantity and quality, highlighting the scarce contribution of fruits, vegetables and protein foods, while sugary foods are excessively high. It should be noted that it is a culture where eating with your hands and from the same container is part of their way of life. This complicates the quantification of consumption rations for each stage of life, as, for the children who are going to enter adolescence and are in full growth, their nutritional needs are not met. In addition, malnutrition affecting height, nutritional deficiencies and multiple infections with frequent intestinal parasites are related to insufficient hygienic measures, water shortages and the difficulty of access to basic food, especially fresh. Thus, since the main problems derive from a lack of adequate hygiene and incorrect nutrition, it is proposed for future editions of the “Holidays in Peace” program, and for field interventions, that sustainable food alternatives and health education programs and nutrition be made available to parents and children. It should even be noted that since the population selected for the program is exposed to very harsh lifestyles, among which is the scarcity of water that is responsible for poor personal and community hygiene, the probability of presenting intestinal parasites is very high, since the route of transmission is oral-fecal. The greater the shortage of hygienic measures, the greater the number of parasitic infections, which aggravates the low caloric intake, thus ending in malnutrition. Increasing programs on hygiene and food education for parents and children in less fortunate regions reduces the risk of infections and health problems. The prevalence of intestinal parasites in Saharawi host children is very high, especially in the case of the protist *G. intestinalis* and *Blastocystis* sp. In most cases, it is multiparasitism combining pathogenic protists with other commensal amoeba or helminths. The combination of direct and indirect diagnostic techniques has allowed the diagnosis of a greater number of cases. Real-time PCR for *G. intestinalis* and conventional PCR for *Blastocystis* sp. have been more sensitive in diagnosis than light microscopy, but microscopy has given us broader species identification. It is convenient to establish a protocol for the diagnosis and treatment of parasites as far as possible because, when they return to their country, reinfections take place; therefore, the objective is to reduce the parasite load and improve the quality of life. With the high prevalence of parasites in children in foster care, empirical treatment of children in foster care is considered appropriate, always under the supervision of visiting pediatricians. All the boys and girls participating in the study present some type of intestinal parasitosis that can be caused by the deficient hygienic sanitary measures of the place. For this reason, hygiene-sanitary education must continue annually to try to change habits that, upon return to their country, can be useful to prevent fecal-oral transmission diseases. A high prevalence of lactose malabsorbers or intolerant participants was found, and those conditions could be a cause of or lead to the aggravation of intestinal parasites, as well as celiac disease. The coexistence of trionomial, malnutrition, intestinal parasites and food intolerances has to be considered for proper nutritional and parasitological management to try to restore children’s health status. All the information of this study will provide us a better point of view of the health status of these children and help to create health education policies for the Saharawi population.

## Figures and Tables

**Table 1:** Percentages for the different types of affectation according to the values of weight, height and BMIZ in the Saharawi children studied.

Z-Score	Height-for-Age Z-Score (HAZ)			Body Mass Index-for-Age Z-Score (BMIZ)		
	Total *n* = 38 (%)	Female *n* = 22 (%)	Male *n* = 16 (%)	Total *n* = 38 (%)	Female *n* = 22 (%)	Male *n* = 16 (%)
<−3	2 (5.3%)	1 (4.5%)	1 (6.3%)	0	0	0
From <−2 to ≥−3	4 (10.5%)	3 (13.6%)	1 (6.3%)	2 (5.3%)	1 (4.5%)	1 (6.3%)
From <−1 to ≥−2	11 (28.9%)	6 (27.3%)	5 (31.3%)	12 (31.6%)	8 (36.4%)	4 (25.0%)
From ≥−1 to ≤+1	21 (55.3%)	12 (54.5%)	9 (56.3%)	24 (63.2)	13 (59.1%)	11 (68.8%)
From ≥+1 to ≤+2				0	0	0
From ≥+2 to ≤+3				0	0	0
>+3	0	0	0	0	0	0

*n* = number of individuals in each group; % = percentage of individuals in each group.

**Table 2:** Foods and food groups according to the FANTA classification [14] consumed by the studied Saharawi child population.

	Total *n* = 38 (%)	Male *n* = 22 (%)	Female *n* = 16 (%)
FARINACEOUS	37 (97.4%)	22 (100%)	15 (93.8%)
Couscous	33 (86.8%)	19 (86.3%)	14 (87.5%)
Rice	37 (97.4%)	22 (100%)	15 (93.8%)
Pasta	8 (21.1%)	5 (19.1%)	3 (18.8%)
Bread	7 (18.4%)	5 (19.1%)	2 (12.5%)
DAIRY PRODUCTS	37 (97.4%)	22 (100%)	15 (93.8%)
Milk	31 (81.6%)	21 (95.5%)	10 (62.5%)
Yogurt	33 (86.8%)	20 (90.9%)	13 (81.3%)
Cheese	6 (15.8%)	5 (19.9%)	1 (6.3%)
LEGUMES	34 (89.5%)	20 (90.9%)	14 (87.5%)
Lentils	33 (86.8%)	20 (90.9%)	13 (81.3%)
Chickpeas	20 (52.6%)	12 (54.5%)	8 (50.0%)
VEGETABLES	0 (0%)	0 (0%)	0 (0%)
FRUITS	31 (81.6%)	19 (86.3%)	12 (75.0%)
PROTEIN FOODS (Meat)	38 (100%)	22 (90.9%)	16 (100%)
OCCASIONAL FOODS	38 (100%)	22 (90.9%)	16 (100%)
Biscuits	37 (97.4%)	22 (90.9%)	5 (31.3%)
Chocolate	33 (86.4%)	18 (81.8%)	15 (93.8%)
Coke beverage	33 (86.4%)	19 (86.3%)	14 (87.5%)
Tea	13 (34.2%)	6 (27.3%)	7 (43.8%)
Candy	38 (100%)	22 (100%)	16 (100%)

*n* = number of individuals in each group; % = percentage of individuals in each group.

**Table 3:** Dietary diversity of the Saharawi child population.

Number of Consumed Food Groups	Total *n* = 38 (%)	Male *n* = 22 (%)	Female *n* = 16 (%)
**1**	3 (7.9%)	2 (9.1%)	1 (6.3%)
**2**	7 (18.4%)	4 (18.2%)	3 (18.8%)
**3**	15 (13.2%)	3 (13.6%)	2 (13.2%)
**4**	18 (47.2%)	11 (50.0%)	7 (47.4%)
**5**	5 (13.2%)	2 (9.1%)	3 (13.2%)

*n* = number of individuals in each group; % = percentage of individuals in each group.

**Table 4:** Frequency of infection/carriage by detected species (protists and helminths) analyzed in the total sample and by sex.

Detected Species	Total *n* = 38 (%)	Male *n* = 2 (%)	Female *n* = 16 (%)
*Giardia intestinalis*	35 (92.1%)	20 (90.9%)	15 (95.8%)
*Blastocystis* sp.	33 (86.8%)	19 (86.4%)	14 (87.5%)
*Endolimax nana*	20 (52.6%)	11 (59.0%)	9 (56.3%)
*Entamoeba coli*	18 (47.4%)	9 (40.9%)	9 (56.3%)
*Entamoeba hartmanni*	14 (36.8%)	6 (27.3%)	8 (50.0%)
*Entamoeba histolytica/E. dispar/E. moshkovskii/E. bangladeshi*	5 (13.2%)	2 (9.1%)	3 (18.8%)
*Iodamoeba butschlii*	5 (13.2%)	3 (13.6%)	2 (12.5%)
*Dientamoeba fragilis*	2 (5.3%)	1 (4.5%)	0 (0)
**Total Parasitization by Protists**	37 (97.4%)	21 (95.5%)	16 (100%)
*Hymenolepis nana*	7 (18.4%)	1 (4.5%)	6 (37.5%)
*Enterobius vermicularis*	4 (10.5%)	2 (9.1%)	2 (12.5%)
**Total Parasitization by Helminths**	10 (26.3%)	3 (13.6%)	7 (43.8%)
**Total Parasitization**	37 (97.4%)	21 (95.5%)	16 (100%)

*n* = number of individuals in each group; % = percentage of individuals in each group.

**Table 5:** Diversity, frequency and molecular features of *Giardia intestinalis* sequences at the *gdh* and *bg* loci obtained in the Saharawi children population under study. GenBank accession numbers are provided.

Gene	Assemblage	Sub-Assemblage	Isolates (*n*)	Reference Sequence	Stretch	Single Nucleotide Polymorphisms	GenBank ID
*gdh*	A	AI	1	L40509	78–484	None	S16
		AII	1	L40510	64–491	None	S6
	B	BIII	1	AF069059	102–394	T147C	S3
			1	AF069059	41–447	C309T	S12
			1	AF069059	40–454	C87Y, G93R, C99T, T147C, G150A, T276Y, C309T, C336Y, G402R	S10
		BIV	1	L40508	74–496	T183C, T387C, C396T, C423T	S15
		BIII/BIV	1	L40508	76–491	C123Y, T135Y, T183C, G186R, C345Y, T366Y, T387C, C396Y, G408R, C423Y, A438R	S2
		BIII/BIV	1	L40508	76–496	T183Y, C255Y, C273Y, C345Y, T366Y, T387C, C396Y, C423Y, A438R	S9
*bg*	A	AII	1	AY072723	141–594	T561C	S16
		B	–	AY072727	97–590	C165T, G180A, C309T, C366T, C450T, C567T	S10
			–	AY072727	102–591	C165T, C309T	S2
			–	AY072727	93–590	C309T	S3
			–	AY072727	102–590	C309Y, G320R, C321Y, C543Y, G552R	S9

R = A/G; Y = C/T.

## References

[1] Besenyö J. (2010). Saharawi refugees in Algeria. AARMS.

[2] Grijalva-Eternod C.S., Wells J.C.K., Cortina-Borja M., Salse-Ubach N., Tondeur M.C., Dolan C., Meziani C., Wilkinson C., Spiegol P., Seal A.J. (2012). The double burden of obesity and malnutrition in a protracted emergency setting: A cross-sectional study of Western Sahara refugees. PLoS Med..

[3] Obokata R., Veronis L., McLeman R. (2014). Empirical research on international environmental migration: A systematic review. Popul. Environ..

[4] Paricio-Talayero J.M., Santos L., Fernández A., Ferriol M., Rodríguez F., Brañas P. (1998). Health examination of children from the Democratic Sahara Republic (Northwest Africa) on vacation in Spain. An. Pediatr..

[5] Lopriore C., Guidoum Y., Briend A., Branca F. (2004). Spread fortified with vitamins and minerals induces catch-up growth and errdicates severe anemia in stunted refugee children aged 3–6 years. Am. J. Clin. Nutr..

[6] Doménech G., Escortell S., Gilabert R., González-Osnaya L., Lucena M., Martínez M.C., Soriano J.M. (2008). Dietary intake and food pattern of Saharawi children refugee in Tindouf (Algeria). Proc. Nutr. Soc..

[7] Soriano J.M., Domènech G., Mañes J., Catalá-Gregori A.I., Barikmo I.E. (2011). Disorders of malnutrition among the Saharawi children. Rev. Esp. Nutr. Hum. Diet..

[8] Bilbao L., Soriano J.M., Doménech G., Martínez C. (2014). La Jaima Alimentaria, guía alimentaria para las familias de acogida de los niños/niñas saharauis. Nutr. Hosp..

[9] Arroyo-Izaga M., Andia V., Demon G. (2016). Diseño de un programa de educación nutricional destinado a mujeres saharauis residentes en los campamentos de Tindouf (Argelia). Nutr. Hosp..

[10] Sarquella G., Asso L., García A.M., Álvarez A. (2004). Use the brief visit for hosted Saharawi children to detect nutritional disorders. An. Pediatr..

[11] Martínez M., Pérez E. Health test for children from Saharawi refugee camps hosted during the summer. Annual Meeting European Society for Social Paediatric.

[12] Soriano J.M., Doménech G., Martínez M.C., Mañes J., Soriano F. (2011). Intestinal parasitic infections in hosted Saharawi children. Trop. Biomed..

[13] Seseña del Olmo G., Rodríguez M.J., Martínez M.C., Pérez J.A. (2011). Prevalencia de parasitosis intestinales en niños de acogida saharauis. Rev. Del Lab. Clin..

[14] WHO Training Course on Child Growth Assessment.

[15] FANTA Introducing the Minimum Dietary Diversity-Women (MDD-W) Global Dietary Diversity Indicator for Women 2014. http://www.fsnnetwork.org/sites/default/files/minimum_dietary_diversity_-_women_mdd-w_sept_2014.pdf.

[16] Estruch R., Ros E., Salas-Salvadó J., Covas M.I., Corella D., Arós F., Gómez-Gracia E., Ruiz-Gutiérrez V., Fiol M., Lapetra J. (2013). Primary prevention of cardiovascular disease with a Mediterranean Diet. N. Engl. J. Med..

[17] Estruch R., Ros E., Salas-Salvadó J., Covas M.I., Corella D., Arós F., Gómez-Gracia E., Ruiz-Gutiérrez V., Fiol M., Lapetra J. (2018). Primari prevention of cadiovascular disease with a Mediterranean Diet supplemented with extra-virgin olive oil or nuts. N. Engl. J. Med..

[18] Verweij J.J., Schinkel J., Laeijendecker D., van Rooyen M.A.A., van Lieshout L., Polderman A.M. (2003). Real-time PCR for detection of Giardia lamblia. Mol. Cell. Probes.

[19] Dacal E., Saugar J.M., de Lucio A., Hernández-de-Mingo M., Robinson E., Köster P.C., Aznar-Ruiz-de-Alegría M.L., Espasa M., Ninda A., Gandasegui J. (2018). Prevalencia and molecular characterization of *Strongyloides stercoralis*, *Giardia duodenalis*, *Cryptosporidium* spp., and *Blastocystis* spp. Isolates in school children in Cubal, Western Angola. Parasites Vectors.

[20] Read C.M., Monis P.T., Thompson R.C. (2004). Discrimination of all genotypes of *Giardia duodenalis* at the glutamate dehydrogenase locus using PCR-RFLP. Infect. Genet. Evol..

[21] Lalle M., Pozio E., Capelli G., Bruschi F., Crotti D., Cacciò S.M. (2005). Genetic heterogeneity at the beta-giardin locus among human and animal isolates of *Giardia duodenalis* and identification of potentially zoonotic subgenotypes. Int. J. Parasitol..

[22] Scicluna S.M., Tawari B., Clark C.G. (2006). DNA barcoding of *Blastocystis*. Protist.

[23] Tamura K., Stecher G., Peterson D., Filipski A., Kumar S. (2013). MEGA6: Molecular Evolutionary Genetics Analysis version 6.0. Mol. Biol. Evol..

[24] Ludvigsson J.F., Bai J.C., Biaqi F., Card T.R., Ciacci C., Ciclitira P.J., Green P.H.R., Hadjisvassiliou M., Holdoway A., van Heel D.A. (2014). Diagnosis and management of adult coeliac disease: Guidelines from the Bristish Society of Gastroenterology. Gut.

[25] Ghosal U. (2011). How to interpret Hydrogen Breath Test. J. Neurogastroenterol. Motil..

[26] Hinojosa-Guadix J.H., Gamarro M.P., Sánchez I.M. (2017). Malabsortion and fructose: Fructose-sorbitol intolerance in functional pathology. Rev. Andal. Patol. Dig..

[27] Sollid L.M., Markussen G., Ek J., Gjerde H., Vartdal F.F., Thorsby E. (1989). Evidence for a primary association of celiac disease to a particular HLA-DQ alpha/beta heterodimer. J. Exp. Med..

[28] Rollan A., Vial C., Quesada S., Espinoza K., Hatton M., Puga A., Repetto G. (2012). Comparative performance of symptoms questionnaire, hydrogen test and genetic test for lactose intolerance. Rev. Med. Chile.

[29] De Onis M. (2004). The use of anthropometry in the prevention of childhood overweight and obesity. Int. J. Obes..

[30] De Onis M., Onyango A.W., Borghi E., Garza E., Yang H. (2006). Comparison of the World Healtth Organitzation (WHO) Child Growth Standerds and the National Center for Health Statistics/WHO international growth reference: Implications for child health programmes. Public Health Nutr..

[31] De Onis M., Onyango A.W., Borghi E., Siyam A., Nishida C., Siekman J. (2007). Development of a WHO growth reference for school-children and adolescents. Bull. World Heath Organ..

[32] Solano L., Acuña I., Barón M., Morón de Salim A., Sánchez A. (2008). Influencia de las parasitosis intestinales y otros antecedentes infecciosos sobre el estado nutricional y antropométrico en niños en situación de pobreza. Parasitol. Latinoam..

[33] Agencia Española de Seguridad Alimentaria y Nutrición (2015). Estudio ALADINO. Estudio de vigilancia del crecimiento, alimentación, actividad física, desarrollo infantil y obesidad en España. http://www.aecosan.msssi.gob.es/AECOSAN/docs/documentos/nutricion/observatorio/Estudio_ALADINO_2015.pdf.

[34] Leone A., Battezzati A., Sara Di Lello S., Ravasenghi S., Mohamed-Iahdih B., Saleh S.M.L., Bertoli S. (2020). Dietary habits of Saharawi type ii diabetic women living in Algerian refugee camps: Relationship with nutritional status and glycemic profile. Nutrients.

[35] Seal A.J., Creeke P.I., Mirghani Z., Abdalla F., McBurney R.P., Pratt L.S., Brookes D., Ruth L.J., Marchand E. (2005). Iron and vitamin A deficiency in long-term African refugee. J. Nutr..

[36] Morseth M., Grewal N., Kaasa I., Harloy A., Barikmo I., Henjum S. (2017). Dietary diversity is related to socioeconomic status among adult Saharawi refugees living in Algeria. BMC Public Health.

[37] UNICEF (United Nations Children’s Foundation) (2008). The Child Care Transition: Innocenti Report Card 8.

[38] Trelis M., Taroncher-Ferrer S., Gozalbo M., Ortiz V., Soriano J.M., Osuna A., Merino-Torres J.F. (2019). *Giardia intestinalis* and fructose malabsorption: A frequent association. Nutrients.

[39] Aakre I., Henjum S., Gjengedal E.L.F., Haugstad C.R., Vollset M., Moubarak K., Ahmed T.S., Alexander J., Kjellevold M., Molin M. (2018). Trace element concentrations in drinking water and urine among saharawi women and young children. Toxics.

[40] Torgerson P.R., de Silva N.R., Fevre E.M., Kasuga F., Rokni M.B., Zhou X.N., Sripa B., Gargouri N., Willingham A.L., Stein C. (2014). The global burden of foodborne parasitic diseases: An update. Trends Parasitol..

[41] Moya-Camarena S.Y., Sotelo N., Valencia M.E. (2002). Effects of asymptomatic *Giardia intestinalis* infection on carbohydrate absorption in well-nourished Mexican children. Am. J. Trop. Med. Hyg..

[42] Grazioli B., Matera G., Laratta C., Schipani G., Guarnieri G., Spiniello E., Imeneo M., Amorosi A., Focà A., Luzza F. (2006). *Giardia lamblia* infection in patients with irritable bowel syndrome and dispepsia: A prospective study. World J. Gastroenterol..

[43] Halliez M.C.M., Buret A.G. (2013). Extraintestinal and long-term consequences of *Giardia duodenalis* infections. World J. Gastroenterol..

[44] Cifre S., Gozalbo M., Ortiz V., Soriano J.M., Merino J.F., Trelis M. (2018). *Blastocystis* subtypes and their association with Irritable Bowel Syndrome. Med. Hypotheses.

[45] Cotton J.A., Beatty J.K., Buret A.G. (2011). Host parasite interactions and phatophysiology in *Giardia* infections. Internat. J. Parasitol..

[46] Sackey M.E., Weigel M.M., Armijos R.X. (2003). Predictors and nutritional consequences of intestinal parasitic infections in rural ecuadorian children. J. Trop. Pediatr..

[47] Ponce-Macotela M., González-Maciel A., Reynoso-Robles R., Martínez-Gordillo M. (2008). Goblets cells: Are they an unspecific barrier against *Giardia intestinalis* or a gate. Parasitol. Res..

[48] Mihai C.M., Balasa A., Mihai L., Stroia V., Stoicescu M.R. (2010). Parasitic infection in children under 2 years old from rural áreas and their relationship with micronutrients deficiencies. Pediatr. Res..

[49] Motta de Oliveira C.L., Ferreira W.A., Da Mata A., Vale-Barbosa M.G. (2011). Anemia of iron deficiency and your correlation with intestinal parasites in a population of the area near urban of Manaus. Rev. Ibero Latinoam. Parasitol..

[50] Feng Y., Xiao L. (2011). Zoonotic potential and molecular epidemiology of *Giardia* species and giardiasis. Clin. Microbiol. Rev..

[51] Skotarczak B. (2018). Genetic diversity and pathogenicity of *Blastocystis*. Ann. Agric. Environ. Med..

[52] Kök M., Çekin Y., Çekin A.H., Uyar S., Harmandar F., Şahintürk Y. (2019). The role of *Blastocystis hominis* in the activation of ulcerative colitis. Turk. J. Gastroenterol..

[53] Gozalbo M. (2012). Estudio Epidemiológico de las Parasitosis Intestinales en Población Infantil del Departamento de Managua (Nicaragua). Ph.D. Thesis.

[54] Crompton D.W.T., Nesheim M.C. (2002). Nutritional impact of intestinal helmintiasis during the human life cycle. Annu. Rev. Nutr..

[55] Sayasone S., Utzinger J., Akkhavong K., Odermatt P. (2015). Multiparasitism and intensity of helmints infections in relation to simptoms and nutritional status among children: A cross-sectional study in southern Lao People’s Democratic Republic. Acta Trop..

[56] Alparo I. (2005). Giardiasis and malnutrition. Rev. Soc. Bol. Ped..

[57] Gasbarrini A., Corazza G.R., Gasbarrini G., Montalto M., Di Stefano M., Basilisco G. (2009). Methodology and indications of H2-breath testing in gastorintestinal diseases: The Rome Consensus Conference. Aliment. Pharmacol. Ther..

[58] Casellas F., Aparici A., Casaus M., Rodríguez P., Malagelada J.R. (2010). subjective perception of lactose intolerance does not always indicate lactose malabsorption. Clin. Gastroentorol. Hepatol..

[59] Chahud A., Zegarre C., Díaz A., Pichilingue O. (1982). Lactose intolerance and giardiasis. Rev. Gastroenterol. Perú..

[60] Rasinperä H., Savilahti E., Enattah N.S., Koukkanen M., Tötterman N., Lindahl H., Järvela i., Kolho K.L. (2004). A genetic test which can be used to diagnose adult-type hypolactasia in children. Gut.

[61] Fasano A., Catassi C. (2001). Current approaches to diagnosis and treatment of celiac disease: An evolving spectrum. Gastroenterology.

[62] Khuffash F.A., Barakat M.H., Shaltout A.A., Farwana S.S. (1987). Coeliac disease among children in Kuwait: Difficulties in diagnosis and management. Gut.

[63] Rawashdah M.O., Khalil B., Raweily E. (1996). Celiac disease in Arabs. J. Pediatr. Gastroenterol. Nutr..

[64] Catassi C., Rätsch I.M., Gandol L., Pratesi R., Fabiani E., Al Asmar R., Frijia M., Bearzi I., Vizzoni L. (1999). Why is celiac disease endemic in the people of the Sahara?. Lancet.

[65] López-Vázquez A. (2004). MHC class I region plays a role in the development of diverse clinical forms of celiac disease in a Saharawi population. Am. J. Gastroenterol..

[66] Rästch I.M., Catassi C. (2007). Coeliac disease: A potentially treatable health problem of Saharawi refugee children. Bull. World Heath Organ..

